# Clinical effects and safety of semi-solid feeds in tube-fed patients: a meta-analysis and systematic review

**DOI:** 10.3389/fnut.2024.1331904

**Published:** 2024-04-25

**Authors:** Limei Feng, Dingchao Xiang, Youping Wu

**Affiliations:** ^1^Department of Neurosurgery, Wuxi No. 5 Affiliated Hospital of Jiangnan University, Wuxi No. 5 People's Hospital, Wuxi, Jiangsu, China; ^2^Department of Neurosurgical Intensive Care Unit, Wuxi Taihu Hospital, Wuxi Clinical College of Anhui Medical University, Wuxi, Jiangsu, China

**Keywords:** enteral nutrition, semi-solid feeds, care, treatment, nursing

## Abstract

**Background:**

Enteral nutrition is a very important form of treatment for critically ill patients. This meta-analysis aimed to evaluate the clinical effects and safety of semi-solid feeds in tube-fed patients.

**Methods:**

Two researchers searched PubMed, clinical trials, Embase, Cochrane Central Register of Controlled Trials, Web of Science, Cochrane Library, China National Knowledge Infrastructure (CNKI), Wanfang Data, and Weipu databases for randomized controlled trials (RCTs) on the clinical effects and safety of semi-solid feeds in tube-fed patients until 10 October 2023. The quality evaluation tool recommended by the Cochrane Library was used to evaluate the quality of included RCTs. RevMan 5.4 software was used for data analysis.

**Results:**

A total of eight RCTs involving 823 tube-fed patients were included in this meta-analysis. A synthesized outcome indicated that semi-solid feeds reduced the incidence of diarrhea (RR = 0.32, 95%CI:0.20–0.50, *P* < 0.001), vomiting (RR = 0.31, 95%CI:0.15–0.64, *P* = 0.002), abdominal distension (RR = 0.41, 95%CI:0.22–0.76, *P* = 0.005), length of intensive care unit (ICU) stay (MD = −3.61, 95%CI: −6.74 to −0.48, *P* = 0.02), and length of hospital stay (MD = −7.14, 95%CI: −10.31 to −3.97, *P* < 0.01) in tube-fed patients. Enteric feeding had no effect on the 30-day mortality (RR = 0.55, 95%CI: 0.19−1.56, *P* = 0.26). No publication bias was detected by the Egger's test results (all *P* > 0.05).

**Conclusion:**

Semi-solid feeds are beneficial in reducing the incidence of diarrhea, abdominal distension, vomiting, and hospital stay. More high-quality studies are needed in the future to verify the effects of semi-solid feeds on mortality.

## Introduction

Enteral nutrition refers to the nutritional support through the gastrointestinal tract to provide various nutrients needed for human metabolism. Enteral nutrition is the best way of nutritional support for critically ill patients, as it has the advantages of protecting gastrointestinal physiological function and the immune barrier and reducing complications of nutrient metabolism and infection (1, 2). Although the use of enteral nutrition is very common, diarrhea, abdominal distension, vomiting, and other gastrointestinal intolerance are usually associated with enteral nutrition, with an incidence of 41.7% to 73.6% (3). In a survey of critically ill surgical patients, it was observed that 13.3% of tube-fed patients were underfed due to gastrointestinal intolerance (4). Feeding intolerance can lead to temporary interruption of enteral nutrition, insufficient nutritional support, prolonged hospital stay, and increased mortality in critically ill patients (5, 6). Therefore, improving the effect and safety of enteral nutrition remains the focus of research in clinical medicine.

Semi-solid feeds such as pectin and other substances are injected through the nasal feeding tube so that pectin and a liquid nutrient solution are mixed and enter the stomach in a semi-solidified chylous state, close to the chylous state as a result of food being ground in the stomach, which is more in line with the needs of the normal human body (7). Some studies have reported that semi-solidified feeding can improve diarrhea and reduce hospitalization time, but due to different intervention objects and intervention schemes, the results are different (8, 9). At present, there are very few systematic review reports on this topic. Therefore, the purpose of this study is to systematically review the reports on the clinical effects and safety of semi-solid feeds in tube-fed patients and further evaluate the role of enteral nutrition through semi-solid feeds on gastrointestinal tolerance in tube-fed patients in order to provide reliable evidence for clinical enteral nutrition practice.

## Methods

This study was conducted and reported in accordance with the Preferred Reporting Items for Systematic reviews and Meta-Analyses (PRISMA) statement (10). Two authors conducted literature review, qualitative research, data extraction, and quality evaluation, respectively, and all the inconsistencies were solved by discussion.

### Strategy for the retrieval of studies from literature

Two researchers searched for randomized controlled trials (RCTs) focused on the clinical effects and safety of semi-solid feeds in tube-fed patients. The searched databases included PubMed, clinical trials, Embase, Cochrane Central Register of Controlled Trials (CENTRAL), Web of Science, Cochrane Library, China National Knowledge Infrastructure (CNKI), Wanfang Data, and Weipu. The search strategies included the following keywords: “pectins” OR “pectinic acid” OR “methoxy pectin” OR “semi-solid” OR “semi–solid nutrients” AND “enteral nutrition” OR “enteral feeding” OR “tube feeding” OR “gastric feeding.” The time limit for the retrieval of data was from the establishment of the database to 10 October 2023. The retrieval strategy adopted the combination of subject words and free words.

### Inclusion and exclusion criteria in literature search

The inclusion criteria for literature search in this study were as follows:

- *Study design*: RCT design on the effect of semi-solid feeds in tube-fed patients. The languages were limited to English and Chinese in the literature search.
- *Participants*: Patients ≥ 18 years old who underwent enteral nutrition solution through nasogastric tube and nasointestinal tube to obtain daily energy.
- *Intervention*: The experimental group was fed with a semi-solidified enteral nutrient solution, and the control group was fed with a routine enteral nutrient solution.
- *Outcome indicators*: The main outcome indicators included the incidence of diarrhea, vomiting, abdominal distension, length of intensive care unit (ICU) stay, length of hospital stay, and 30-day mortality.

We excluded reviews, case reports, conference abstracts, editorials, and comments from the search.

### Quality assessment

This meta-analysis used the quality evaluation tool recommended by Cochrane Library to evaluate the quality of the retrieved literature. The tool mainly includes the following seven aspects: (i) the random allocation method; (ii) the hidden allocation scheme; (iii) the blind method for subjects and implementers of treatment; (iv) the blind method for outcome assessment; (v) integrity of data; (vi) selective reporting of research results; and (vii) other sources of bias. Literature screening and quality evaluation were completed independently by two researchers.

### Data extraction

The following contents were extracted by two authors: the first author's name, publication year, study setting, sample size, participants' average age, details of tube feeding intervention, duration of intervention, outcome indicators, and conclusion of the study. All disagreements were resolved by consensus.

### Statistical analysis

This meta-analysis used RevMan 5.4 software for data analysis. Discontinuous variables were pooled with relative risk (RR) and 95% confidence interval (CI), and the continuous variables were pooled with mean difference (MD) and 95% CI. In this study, the chi-square test was used to analyze the heterogeneity of the results. If I^2^ is ≤ 50% and P is ≥0.1, it was determined that there was no heterogeneity among the studies and a fixed-effect model was used for meta-analysis. If I^2^ is >50% and P is 12 1 < 0.01, it was determined that there was heterogeneity among the studies and a random effect model was used for analysis. Egger's test and funnel plots were performed to evaluate the potential publication bias. A *p*-value of < 0.05 was considered to show statistically significant difference between groups.

## Results

### Selection of RCTs

**Figure 1:**
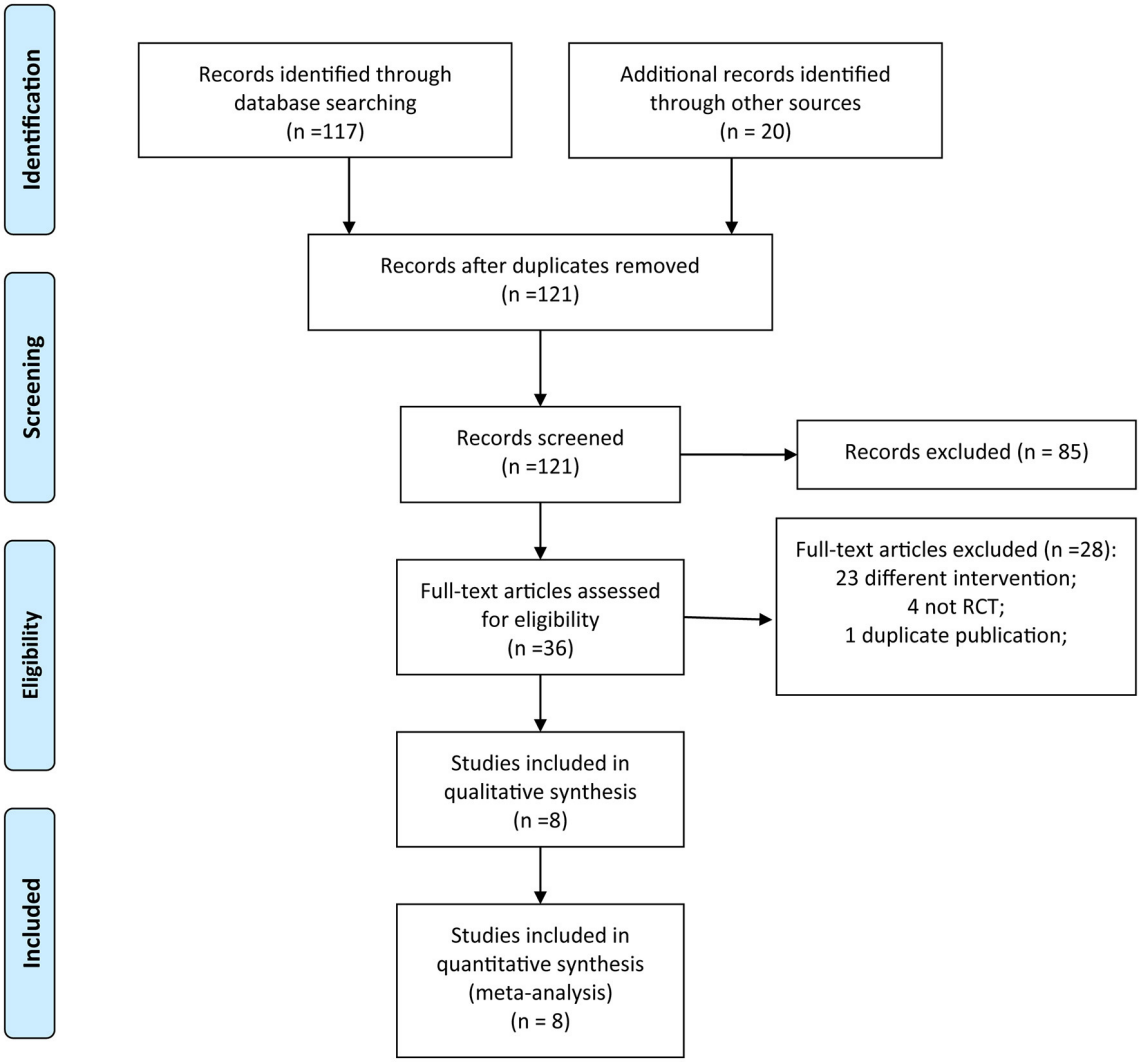
Flowchart of RCT selection.

In this meta-analysis, 121 reports were identified after duplications were removed. After screening the titles and abstracts, 85 studies were excluded. The full texts of the remaining 36 articles were evaluated. Twenty-eight articles were excluded after reading the full text based on the inclusion and exclusion criteria. Finally, eight RCTs (11–18) were included in this meta-analysis (Figure 1).

### Characteristics of RCTs

As presented in Table 1, in the included eight RCTs, there were a total of 823 tube-fed patients, of which 397 patients were given semi-solid feeds and 426 patients were given the traditional feeding. The included RCTs were reported from China and Japan, most included patients were older than 60 years old, and the duration of the intervention differed from 3 days to 2 weeks.

**Table 1:** Characteristics of included RCTs.

**RCT**	**Country**	**Sample size**		**Age (years)**		**Intervention**		**Daily average dose of enteral nutrition**		**Enteral nutrition formulas**		**Duration of intervention**
		**Semi-solid feeds group**	**Control group**	**Semi-solid feeds group**	**Control group**	**Semi-solid feeds group**	**Control group**	**Semi-solid feeds group**	**Control group**	**Semi-solid feeds group**	**Control group**	
Lu et al. (11)	China	14	14	54.93 ± 20.96	52.14 ± 13.77	Intermittent feeding of pectin and nutrient solution	Intermittent feeding of nutrient solution	100–300 mL	100–300 mL	Pectin 60 mL was before EN	Whole enteral feeding preparation	3 days
Maruyama et al. (12)	Japan	98	100	77.9 ± 11.3	79.5 ± 10.9	Intermittent feeding of pectin and nutrient solution	Intermittent feeding of nutrient solution	20–30 kcal/d/kg	20–30 kcal/d/kg	Pectin-containing oligomeric formula containing 9 mg/kcal pectin	Standard polymeric formula	1 week
Shao et al. (13)	China	46	47	64.74 ± 10.11	66.12 ± 10.44	Intermittent feeding of pectin and nutrient solution	Intermittent feeding of nutrient solution	20–25 kcal/d	20–25 kcal/d	Pectin and Enteral nutrition was proportioned according to the calcium content of enteral nutrition preparation (pectin: Ruineng = 1:5)	Whole enteral feeding preparation: Ruineng	1 week
Tabei et al. (14)	Japan	15	12	82.7	82.9	Intermittent feeding of pectin and nutrient solution	Intermittent feeding of nutrient solution	300–400 kcal	300–400 kcal	Viscosity-regulating pectin solution (1.4 g fiber, 120 mg sodium, and 87.8 g water per bag (90 g, 5 kcal)	The liquid EN diet containing 3.5 g protein, 3.3 g fat, 14.1 g carbohydrate, and other essential macronutrients and micronutrients per 100 mL (100 kcal)	2 weeks
Toh et al. (15)	Japan	45	72	80.7	81	Intermittent feeding of pectin and nutrient solution	Intermittent feeding of nutrient solution	Not reported	Not reported	PG Soft with dynamic viscosity 20,000 mPa·s	Liquid feed (containing 4 g protein, 2.8 g fat, 14.5 g carbohydrate, and 1 g dietary fiber per 100 kcal)	2 weeks
Wang (16)	China	55	55	64.74 ± 10.11	65.01 ± 10.10	Intermittent feeding of pectin and nutrient solution	Intermittent feeding of nutrient solution	20–25 kcal/d	20–25 kcal/d	Soluble dietary fiber (pectin), mix pectin and nutrient solution according to 1:5	Enteral nutrition suspension (1.5 kcal/mL)	2 weeks
Xi et al. (17)	China	62	63	48.7 ± 10.7	48.2 ± 13.7	Intermittent feeding of pectin and nutrient solution	Intermittent feeding of nutrient solution	25–30 kcal/d	25–30 kcal/d	The nutritional support program was the same as EN group except that an additional amount of pectin was administrated once 4 h ahead of EN	5% glucose at a rate of 25 mL/h was given on day 1, followed with an initial amount of EN (31.3 g peptisorb dissolved in 250 mL water) at 12.5 mL/h on day 2. From day 3 to day 6, EN with 62.5 g peptisorb dissolved in 250 mL water was administrated at 12.5 mL/h.	1 week
Zang et al. (18)	China	62	63	63.6 ± 9.9	61.6 ± 11.4	Intermittent feeding of pectin and nutrient solution	Intermittent feeding of nutrient solution	20–25 kcal/d	20–25 kcal/d	Rifampicin pectin 90 mL was before Ruineng EN	Whole enteral feeding preparation: Ruineng	1 week

### Quality of RCTs

**Figure 2:**
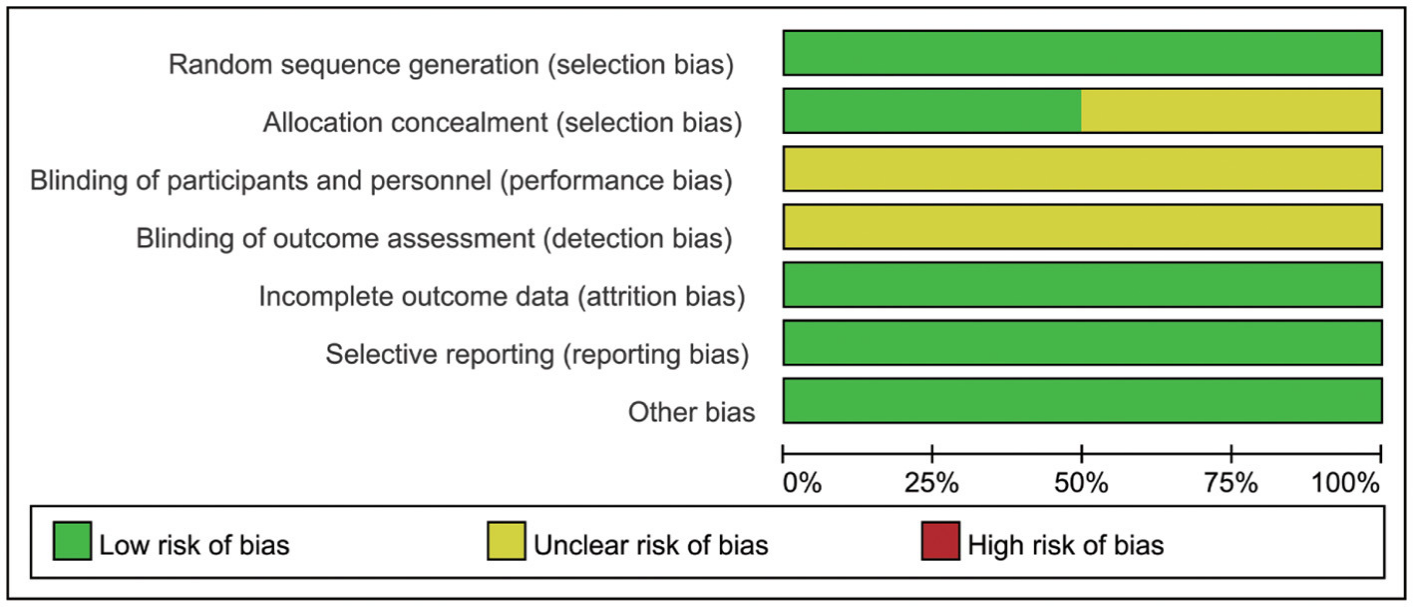
Risk of bias graph.

**Figure 3:**
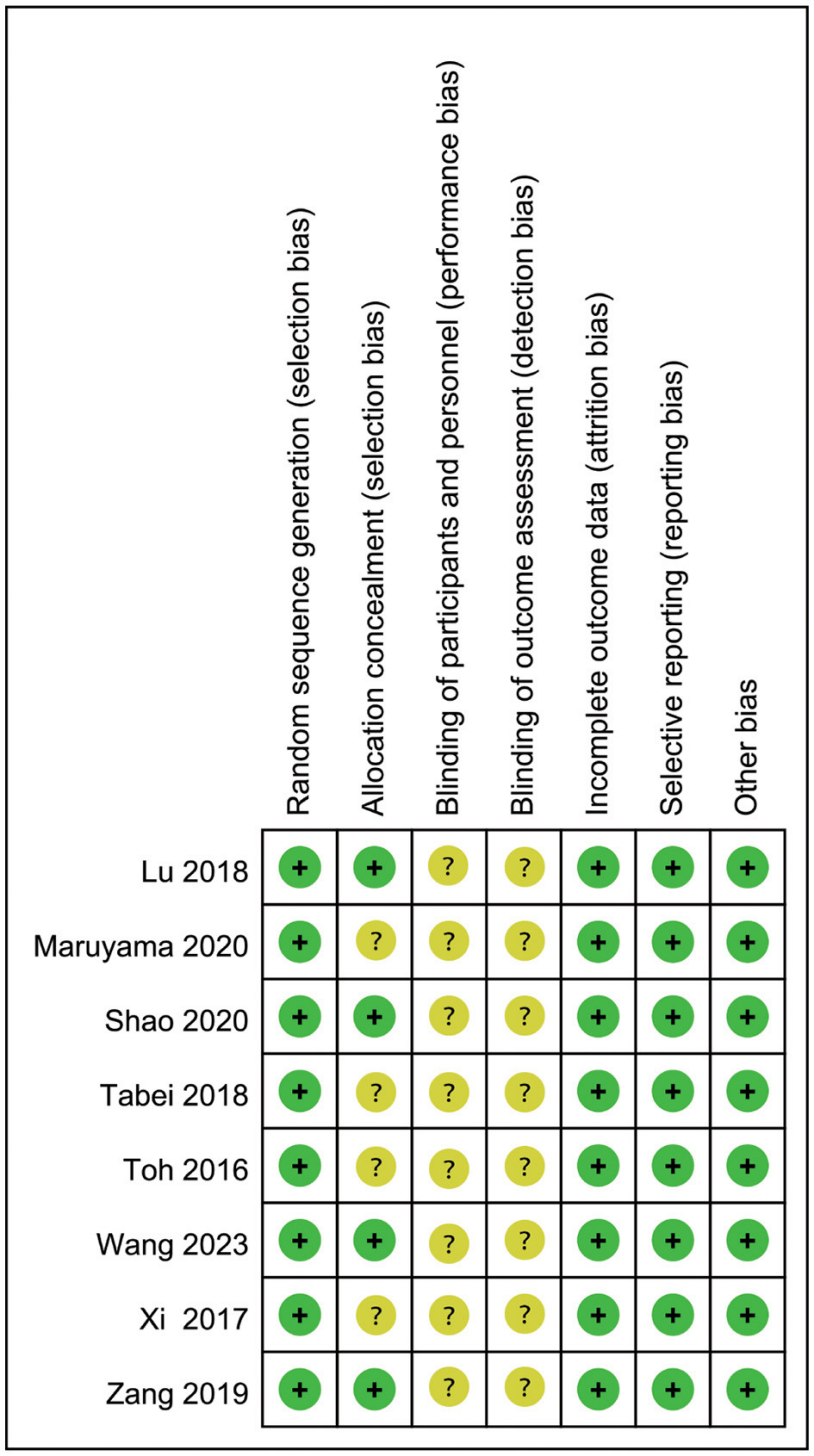
Risk of bias summary.

As shown in Figures 2 and 3, although all the included studies were RCTs, the major factor influencing the quality was that participants, intervention personnel, and the outcome evaluator were not blinded, which might have a subjective influence on judging the outcome, leading to the findings of a positive trend. No other risk of biases was found.

### Meta-analysis

**Figure 4:**
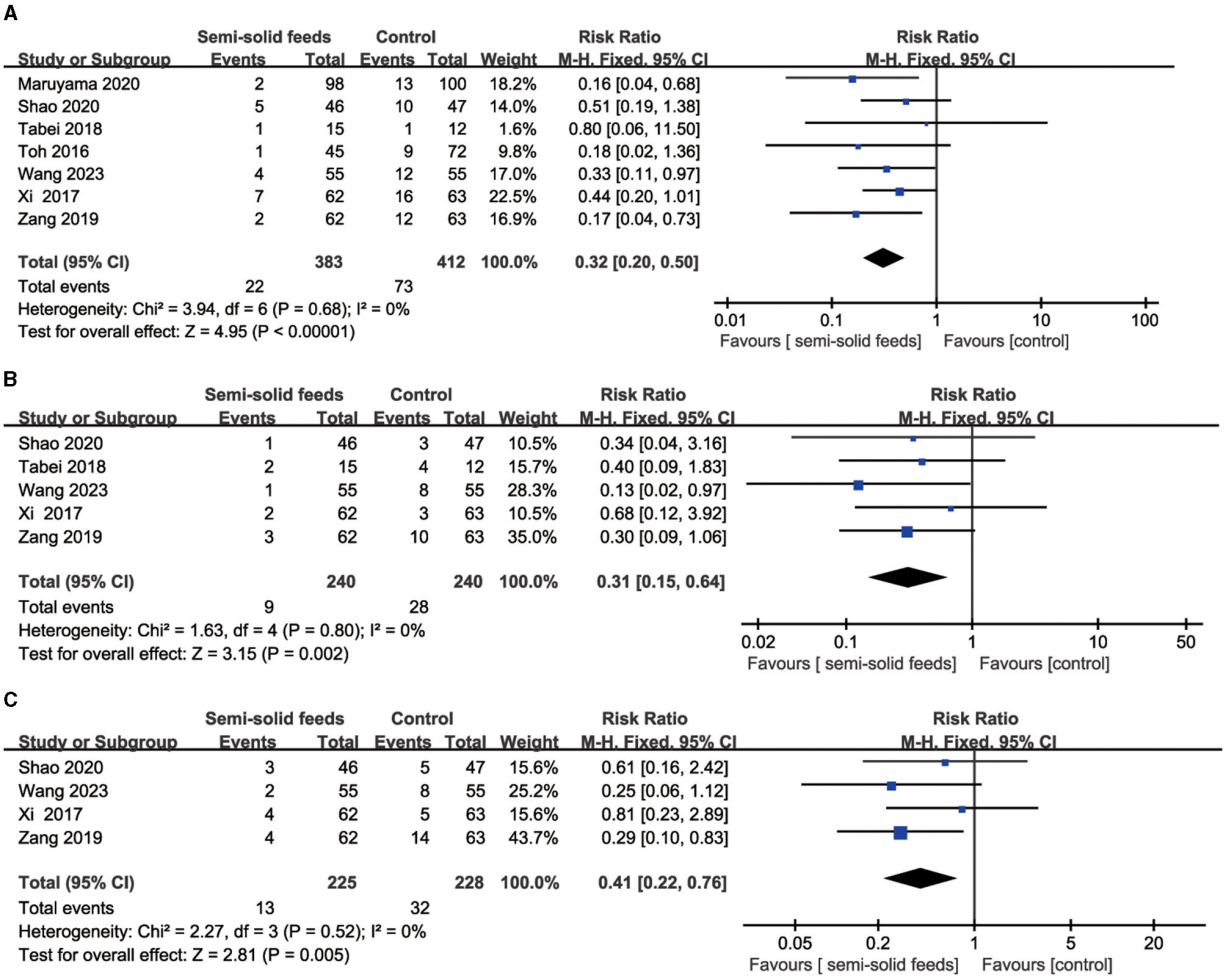
Forest plots for the incidence of diarrhea, vomiting, and abdominal distension. **(A)** Forest plot for the incidence of diarrhea. **(B)** Forest plot for the incidence of vomiting. **(C)** Forest plot for the incidence of abdominal distension.

Seven RCTs reported the incidence of diarrhea. No heterogeneity (I^2^ = 0%, *P* = 0.68) was found in this outcome, and fixed model was used for data analysis. A synthesized outcome indicated that semi-solid feeds reduced the incidence of diarrhea in tube-fed patients (RR = 0.32, 95%CI:0.20−0.50, *P* < 0.001, Figure 4A).

Five RCTs reported the incidence of vomiting. No heterogeneity (I^2^ = 0%, *P* = 0.80) was found in this outcome and fixed model was used for data analysis. A synthesized outcome indicated that semi-solid feeds reduced the incidence of vomiting in tube-fed patients (RR = 0.31, 95%CI: 0.15−0.64, *P* = 0.002, Figure 4B).

Four RCTs reported the incidence of abdominal distension. No heterogeneity (I^2^ = 0%, *P* = 0.52) was found in this outcome, and fixed model was used for data analysis. A synthesized outcome indicated that semi-solid feeds reduced the incidence of abdominal distension in tube-fed patients (RR = 0.41, 95%CI:0.22−0.76, *P* = 0.005, Figure 4C).

As presented in Table 2, this meta-analysis found that semi-solid feeds reduced the length of ICU stay (MD = −3.61, 95%CI: −6.74 to −0.48, *P* = 0.02) and length of hospital stay (MD = −7.14, 95%CI: −10.31 to −3.97, *P* < 0.01) in tube-fed patients. Enteral feeding was found to have no effect on the 30-day mortality (RR = 0.55, 95%CI: 0.19−1.56, *P* = 0.26).

**Table 2:** Meta-analysis results of length of ICU stay, length of hospital stay, and 30-day mortality.

**Outcome**	**Number of included RCTs**	**I^2^**	**Model for meta-analysis**	**RR/MD**	**95%CI**	***P***
Length of ICU stay	2	44	Fixed	−3.61	−6.74 to −0.48	0.02
Length of hospital stay	2	0	Fixed	−7.14	−10.31 to −3.97	<0.01
30-Day mortality	3	5	Fixed	0.55	0.19–1.56	0.26

RCT, randomized controlled trial; ICU, intensive care unit; RR, relative risk; MD, mead difference; CI, confidence interval.

### Sensitivity analysis

We excluded the individual studies included one by one for sensitivity analysis, and the results showed that the combined effects of each study did not change significantly, indicating that the meta-analysis results of this study were stable and reliable.

### Publication bias

**Figure 5:**
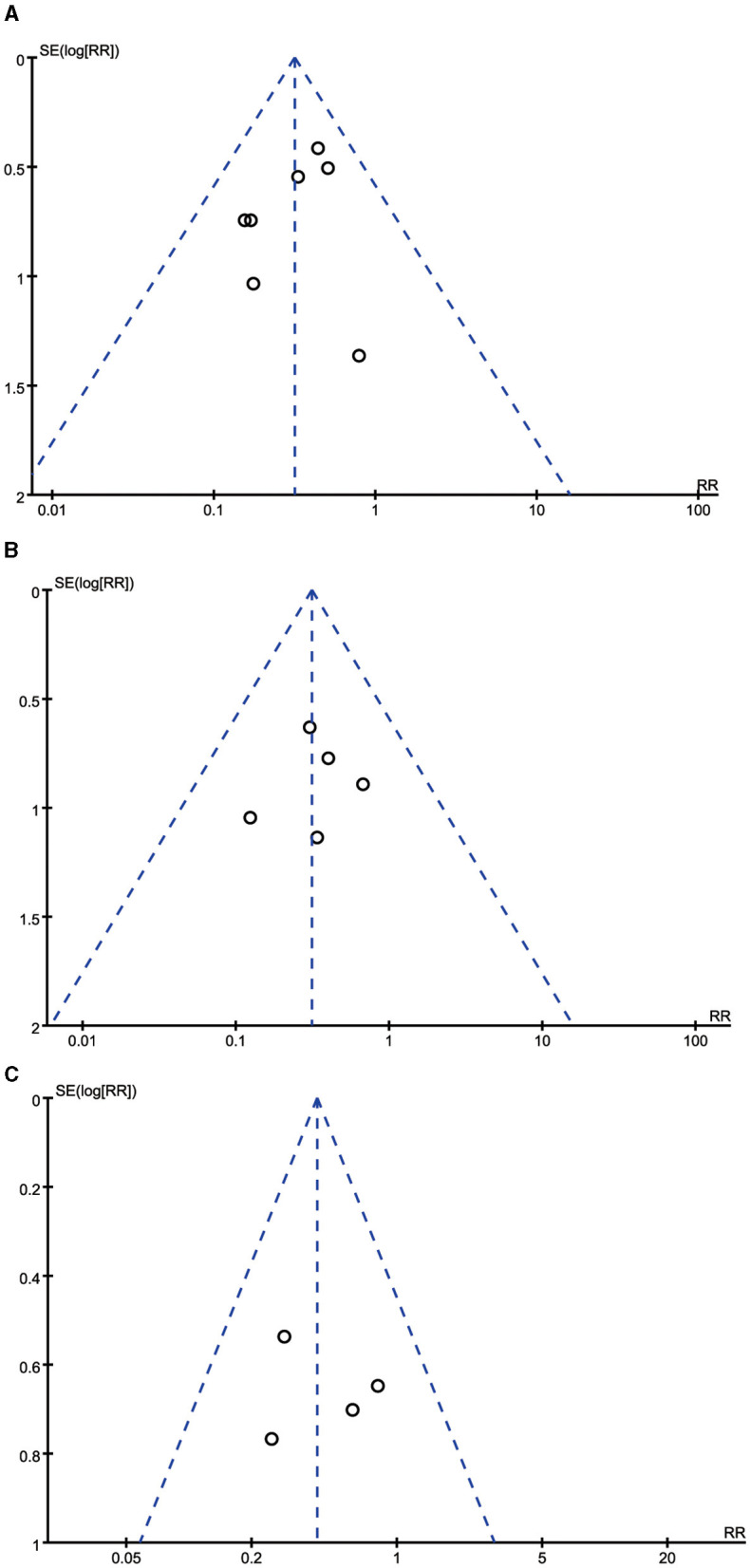
Funnel plots for the incidence of diarrhea, vomiting, and abdominal distension. **(A)** Funnel plot for the incidence of diarrhea. **(B)** Funnel plot for the incidence of vomiting. **(C)** Funnel plot for the incidence of abdominal distension.

As shown in Figure 5, the dots in the funnel plots were evenly distributed. Moreover, no publication bias was detected by the Egger's test results (all *P* > 0.05).

## Discussion

Enteral nutrition through semi-solid feeds means that the solution containing pectin, the semi-curing agent, and the enteral nutrition solution are fed successively, and they are all in a liquid state before feeding. After feeding, pectin will be mixed with the enteral nutrition solution containing free calcium ions to reach a semi-solidified state under the acidic condition in the stomach (19). Enteral nutrition through semi-solid feeds have the advantages of simple preparation and easy operation (20). On the basis of traditional enteral nutrition solution, semi-curing agents such as pectin, agar, and guar gum are added to fuse it into semi-solidified chyli in the stomach or *in vitro*, which is similar to the chyli ground by stomach and close to the normal physiological diet state of human body (21–23). It is beneficial for digestion and absorption by the human body so as to prevent the occurrence of enteral feeding intolerance. The results of this meta-analysis have found that semi-solid feeds are beneficial to reduce the incidence of diarrhea, vomiting, abdominal distension, length of ICU stay, and length of hospital stay, which is consistent with the findings of a previous meta-analysis (24).

Diarrhea is the most common symptom of enteral feeding intolerance in critically ill patients during enteral nutrition, with an incidence of 30.8%. Diarrhea in critically ill patients will reduce the absorption of nutrients and secondary water, cause electrolyte balance disorders and skin and mucous membrane damage, and increase the risk of infection and death (25). In addition, it will also affect the psychological state of patients and increase the workload of nursing (26). Previous studies (24, 27) have shown that enteral nutrition through semi-solid feeds can reduce the incidence of poor nutrition and diarrhea in critically ill patients. The improvement of diarrhea in critically ill patients is mainly related to the addition of pectin as a semi-curing agent during enteral nutrition (28). Dietary fiber can protect the immune barrier function of the gastrointestinal tract, improve the tolerance of the gastrointestinal tract, and promote human health, and pectin is an important soluble dietary fiber (29). On the one hand, pectin can activate or inhibit the response of dendritic cells and macrophages, stimulate the diversity and richness of beneficial microbial communities, and enhance the immune barrier function of gastrointestinal tract by promoting the adhesion of symbiotic bacteria and inhibiting the adhesion of pathogens to epithelial cells (30). After the short-chain fatty acids decomposed by pectin in the intestine are absorbed by the colon, it will increase the Na^+^ levels and water absorption in intestinal mucosa and reduce the water content of feces, thus reducing the incidence of diarrhea (31). Semi-solid feeds can lower the incidence of diarrhea in critically ill patients during enteral nutrition, but whether it will aggravate the occurrence of gastric retention has not been reported, which needs to be further investigated in the future (32).

This meta-analysis found that semi-solid feeds can reduce the incidence of vomiting. There are many reasons for vomiting, one of which is that critically ill patients are prone to gastrointestinal dysfunction and decreased gastric motility. The common nutrient solution is dilute liquid. Critically ill patients are more likely to have gastric reflux when they are lying on their back, resulting in vomiting (33). Pectin can combine well with calcium ions in the nutrient solution without changing the composition of the nutrient solution to form semi-solid, which is similar to the chyme state in which food is ground in the stomach and reduces the occurrence of reflux (34, 35). Reducing the incidence of vomiting is more in line with the physiological characteristics of human digestion and absorption.

Critically ill patients will develop abdominal distension in the process of receiving enteral nutrition, which showed an incidence of 26.9–43.8% in a study (36). After abdominal distension occurs in critically ill patients, on the one hand, flatulence will oppress the diaphragm and chest, resulting in vomiting, poor appetite, dyspnea, and interruption of enteral nutrition, seriously affecting their treatment and rehabilitation (37). On the other hand, it will increase intraperitoneal pressure, obstruction of inferior vena cava reflux, and insufficient blood perfusion of the abdominal organs, resulting in venous thrombosis of lower extremities and acute injury of abdominal organs (38). The serious condition of critically ill patients, the weakening of gastrointestinal motility, and the increase of intestinal bacteria are the important causes of abdominal distension (39). Enteral nutrition through a semi-solidified substance containing pectin can inhibit the sudden flow of nutrients from the stomach into the duodenum, avoid the inhibition of the duodenum on gastric movement, enhance gastric peristalsis, and thus reduce the occurrence of abdominal distension (40). Pectin can be decomposed into short-chain fatty acids in the intestinal tract, reducing the pH in the intestinal tract and increasing the number of probiotics in the intestinal tract, while probiotics can improve the intestinal blood supply and enhance intestinal peristalsis, thus reducing the occurrence of abdominal distension (41, 42). Critically ill patients on enteral nutrition support for necessary nutrients often cannot consume the required calories on time, which will seriously affect their nutritional support and hinder improvement of their nutritional status, thereby prolonging the length of hospital stay of patients. On the one hand, semi-solid feeds can shorten the time for critically ill patients to reach the standard of nutrition and improve the required calorific intake so as to provide guarantee for their rehabilitation. On the other hand, semi-solid feeds can protect the immune barrier function of the gastrointestinal tract, reduce the occurrence of infectious diseases, and shorten the duration of hospitalization. However, when enteral nutrition is semi-solidified, attention should be paid to investigating whether there is a pharmacokinetic interaction between enteral nutrition and some drugs that are in use (43). In this study, we have found that semi-solid feeds have no significant effect on reducing 30-day mortality. It may be because of the variation in the severity of the disease of the included patients, fewer number of studies included in this meta-analysis, and limitations of our conclusion. More follow-up RCTs with larger sample size in the role of semi-solid feeds on mortality are needed.

At present, two methods of enteral nutrition (intermittent feeding and continuous feeding) that are usually adopted have their advantages and disadvantages. Most of the included RCTs in this meta-analysis have used intermittent enteral nutrition infusions. Intermittent feeding can establish a pattern of intermittent secretion of gastrointestinal hormones, which is more conducive to the establishment of a basic physiological environment for digestion and absorption in the gastrointestinal tract (44–46). A study has shown that intermittent feeding can reduce the number of bacteria in the stomach, especially at night. Because the pH value in the stomach is not affected by eating, it can ensure effective blood perfusion of the gastrointestinal mucosa and prevent and cure intestinal bacterial translocation (47). However, some studies have found that intermittent feeding without infusion pump leads to a higher incidence of gastric tube dislocation, aspiration pneumonia, and abdominal distension than continuous infusion (48, 49). Although there is no periodic fluctuation of gastrointestinal hormones during intermittent feeding, continuous enteral nutrition can maintain insulin, gastrin, and other gastrointestinal hormones at a high level, which is beneficial to intestinal absorption (50). Furthermore, it has been found that early enteral nutrition for trauma patients in the ICU is associated with less wound infection, lower mortality, and shorter hospital stay (51). Moreover, early enteral nutrition is safe and well-tolerated and can reduce the in-hospital mortality of patients receiving extracorporeal membrane oxygenation (52). Therefore, the role of semi-solid feeds for intermittent vs. continuous enteral nutrition infusions and early vs. delayed enteral nutrition needs to be further investigated in the future.

This present meta-analysis has several limitations. First, this meta-analysis only has searched the published literature in Chinese and English and did not include gray literature, which may have led to the omission of some relevant studies. Second, the formula—the use of pectin, input speed, and time—is not uniform in the RCTs considered in this meta-analysis, which is also one of the reasons for its heterogeneity. Third, some of these studies have been done inside ICU and some outside of it. Finally, it has been reported that early enteral nutrition is related to improved outcomes in critically ill, mechanically ventilated, medical and surgical patients (53). Early initiation of enteral nutrition vs. delayed enteral nutrition may have different prognostic outcomes. Most of the included RCTs do not report the initiation time of enteral nutrition. Furthermore, the RCTs included in this meta-analysis cover patients with different types of diseases, which may increase the heterogeneity of study population and create bias in the results; thus, our findings should be treated with caution. Therefore, in the future, it is necessary to carry out large-sample and high-quality RCTs to further explore the efficacy and safety of semi-solid feeds for enteral nutrition so as to provide more reliable evidence for clinical treatment and care.

## Conclusion

In summary, the results of this meta-analysis have indicated that semi-solid feeds can reduce the incidence of diarrhea, abdominal distension, and vomiting and reduce the length of ICU and hospital stay, but sufficient evidence is lacking to support the effects of semi-solid feeds on reducing 30-day mortality. Relevant guidelines or scientific guidance recognized by experts on the semi-solid feeds is lacking. In the future, it is necessary to investigate the effects and safety of semi-solid feeds for enteral nutrition on the incidence of gastric retention, constipation, and pharmacokinetic effects with other drugs.

## Data availability statement

The original contributions presented in the study are included in the article/supplementary material, further inquiries can be directed to the corresponding author.

## Author contributions

LF: Data curation, Investigation, Methodology, Writing – original draft, Writing – review & editing. DX: Investigation, Writing – original draft. YW: Investigation, Writing – original draft.

## References

[1] Doley J. Enteral nutrition overview. Nutrients. (2022) 14:2180. 10.3390/nu14112180 35683980 PMC9183034

[2] Tripodi SI Bergami E Panigari A Caissutti V Brovia C De Cicco M . The role of nutrition in children with cancer. Tumori. (2023) 109:19–27. 10.1177/03008916221084740 35722985 PMC9896537

[3] Preiser JC Arabi YM Berger MM Casaer M McClave S Montejo-Gonzalez JC . A guide to enteral nutrition in intensive care units: 10 expert tips for the daily practice. Crit Care. (2021) 25:424. 10.1186/s13054-021-03847-4 34906215 PMC8669237

[4] Peev MP Yeh DD Quraishi SA Osler P Chang Y Gillis E . Causes and consequences of interrupted enteral nutrition: a prospective observational study in critically ill surgical patients. J Parenter Enteral Nutr. (2015) 39:21–7. 10.1177/0148607114526887 24714361 PMC4402286

[5] Sun JK Nie S Chen YM Zhou J Wang X Zhou SM . Effects of permissive hypocaloric vs standard enteral feeding on gastrointestinal function and outcomes in sepsis. World J Gastroenterol. (2021) 27:4900–12. 10.3748/wjg.v27.i29.4900 34447234 PMC8371509

[6] Arabi YM Aldawood AS Haddad SH Al-Dorzi HM Tamim HM Jones G . Permissive underfeeding or standard enteral feeding in critically ill adults. N Engl J Med. (2015) 372:2398–408. 10.1056/NEJMoa1502826 25992505

[7] Kokura Y Suzuki C Wakabayashi H Maeda K Sakai K Momosaki R. Semi-solid nutrients for prevention of enteral tube feeding-related complications in japanese population: a systematic review and meta-analysis. Nutrients. (2020) 12:1687. 10.3390/nu12061687 32516973 PMC7353039

[8] Issaka AI Agho KE Page AN Burns P Stevens GJ Dibley MJ. Determinants of early introduction of solid, semi-solid or soft foods among infants aged 3-5 months in four Anglophone West African countries. Nutrients. (2014) 6:2602–18. 10.3390/nu6072602 25025297 PMC4113759

[9] Kanie J Suzuki Y Akatsu H Kuzuya M Iguchi A. Prevention of late complications by half-solid enteral nutrients in percutaneous endoscopic gastrostomy tube feeding. Gerontology. (2004) 50:417–9. 10.1159/000080181 15477704

[10] Page MJ McKenzie JE Bossuyt PM Boutron I Hoffmann TC Mulrow CD . The PRISMA 2020 statement: an updated guideline for reporting systematic reviews. BMJ. (2021) 372:n71. 10.1136/bmj.n71 33782057 PMC8005924

[11] Lu K Zeng F Li Y Chen C Huang M. A more physiological feeding process in ICU: Intermittent infusion with semi-solid nutrients (CONSORT-compliant). Medicine. (2018) 97:e12173. 10.1097/MD.0000000000012173 30200118 PMC6133414

[12] Maruyama M Goshi S Kashima Y Mizuhara A Higashiguchi T. Clinical effects of a pectin-containing oligomeric formula in tube feeding patients: a multicenter randomized clinical trial. Nutr Clin Pract. (2020) 35:464–70. 10.1002/ncp.10392 31606903

[13] Shao X Lin Z Li Y. Effect of semi-solidified intermittent enteral nutrition on reducing enteral nutrition intolerance in critically ill patients. Nurs J People Liber Army. (2020) 37:60–6.

[14] Tabei I Tsuchida S Akashi T Ookubo K Hosoda S Furukawa Y . Effects of a novel method for enteral nutrition infusion involving a viscosity-regulating pectin solution: a multicenter randomized controlled trial. Clin Nutr ESPEN. (2018) 23:34–40. 10.1016/j.clnesp.2017.11.005 29460811

[15] Toh Yoon EW Yoneda K Nishihara K. Semi-solid feeds may reduce the risk of aspiration pneumonia and shorten postoperative length of stay after percutaneous endoscopic gastrostomy (PEG). Endosc Int Open. (2016) 4:E1247–51. 10.1055/s-0042-117218 27995184 PMC5161128

[16] Wang Y. Effect of semi-solidified intermittent enteral nutrition on critically ill patients in intensive care unit. Chin Commun Phys. (2023) 39:50–52.

[17] Xi F Xu X Tan S Gao T Shi J Kong Y . Efficacy and safety of pectin-supplemented enteral nutrition in intensive care: a randomized controlled trial. Asia Pac J Clin Nutr. (2017) 26:798–803. 28802288 10.6133/apjcn.082016.07

[18] Zang L Shi M Zhang X. Application of pectin plus intermittent enteral nutrition infusion in stroke patients with dysphagia. J Nurs. (2019) 26:55–8.

[19] Pascale N Gu F Larsen N Jespersen L Respondek F. The potential of pectins to modulate the human gut microbiota evaluated by in vitro fermentation: a systematic review. Nutrients. (2022) 14:3629. 10.3390/nu14173629 36079886 PMC9460662

[20] Elshahed MS Miron A Aprotosoaie AC Farag MA. Pectin in diet: interactions with the human microbiome, role in gut homeostasis, and nutrient-drug interactions. Carbohydr Polym. (2021) 255:117388. 10.1016/j.carbpol.2020.117388 33436217

[21] Jiao J Chen Y Yang L Li W Zhou Z Li L . Nursing practice based on evidence-based concepts to prevent enteral nutrition complications for critically ill neurosurgical patients. Front Surg. (2022) 9:857877. 10.3389/fsurg.2022.857877 35372491 PMC8971188

[22] Nakagawa M Sugihara K Isobe K Akasu M Tsujimoto K Itsui Y . A case of tracheal obstruction caused by reflux and aspiration of semi-solid nutrients via the nasogastric tube. Int J Surg Case Rep. (2019) 65:217–20. 10.1016/j.ijscr.2019.11.004 31733618 PMC6864173

[23] Arabi YM. Predicting enteral feeding intolerance in patients with sepsis: why and how? Saudi J Gastroenterol. (2022) 28:1–2. 10.4103/sjg.sjg_38_22 35083976 PMC8919924

[24] Li C Shen M. Meta-analysis of the effect of semi-solidification of enteral nutrition on gastrointestinal tolerance in patients with tube feeding. Chin J Nurs. (2021) 28:5–9.

[25] Ni W Jiao X Zou H Jing M Xia M Zhu S . Gut microbiome alterations in ICU patients with enteral nutrition-related diarrhea. Front Microbiol. (2022) 13:1051687. 10.3389/fmicb.2022.1051687 36483214 PMC9722739

[26] Xie Y Tian R Wang T Jin W Hou Y Zhou Z . A prediction model of enteral nutrition complicated with severe diarrhea in ICU patients based on CD55. Ann Palliat Med. (2021) 10:1610–9. 10.21037/apm-20-1050 33222452

[27] Li Y Hou L Jiang E. Application and nursing of semi-solidified feeding in enteral nutrition of critically ill patients. Milit Nurs. (2023) 40:93–96.

[28] Cresswell JA Ganaba R Sarrassat S Cousens S Some H Diallo AH . Predictors of exclusive breastfeeding and consumption of soft, semi-solid or solid food among infants in Boucle du Mouhoun, Burkina Faso: a cross-sectional survey. PLoS ONE. (2017) 12:e0179593. 10.1371/journal.pone.0179593 28640900 PMC5480894

[29] Nakayama T Hayashi S Okishio K Tomishiro T Hosogai K Ootsu Y . Prompt improvement of a pressure ulcer by the administration of high viscosity semi-solid nutrition via a nasogastric tube in a man with tuberculosis: a case report. J Med Case Rep. (2010) 4:24. 10.1186/1752-1947-4-24 20205856 PMC2825516

[30] Blanco-Perez F Steigerwald H Schulke S Vieths S Toda M Scheurer S. The dietary fiber pectin: health benefits and potential for the treatment of allergies by modulation of gut microbiota. Curr Allergy Asthma Rep. (2021) 21:43. 10.1007/s11882-021-01020-z 34505973 PMC8433104

[31] Khongcharoensombat T Khemtong A Lakananurak N. Pectin-containing compared with standard polymeric formula in enteral nutrition: a randomized controlled parallel study in Thailand. Asia Pac J Clin Nutr. (2021) 30:67–74. 33787042 10.6133/apjcn.202103_30(1).0009

[32] Hu W Cassard AM Ciocan D. Pectin in metabolic liver disease. Nutrients. (2022) 15:157. 10.3390/nu15010157 36615814 PMC9824118

[33] Nakamura K Inokuchi R Fukushima K Naraba H Takahashi Y Sonoo T . Pectin-containing liquid enteral nutrition for critical care: a historical control and propensity score matched study. Asia Pac J Clin Nutr. (2019) 28:57–63. 30896415 10.6133/apjcn.201903_28(1).0009

[34] Pedrosa LF Raz A Fabi JP. The complex biological effects of pectin: galectin-3 targeting as potential human health improvement? Biomolecules. (2022) 12:289. 10.3390/biom12020289 35204790 PMC8961642

[35] Dang G Wang W Zhong R Wu W Chen L Zhang H. Pectin supplement alleviates gut injury potentially through improving gut microbiota community in piglets. Front Microbiol. (2022) 13:1069694. 10.3389/fmicb.2022.1069694 36569061 PMC9780600

[36] Wanden-Berghe C Patino-Alonso MC Galindo-Villardon P Sanz-Valero J. Complications associated with enteral nutrition: CAFANE study. Nutrients. (2019) 11:2041. 10.3390/nu11092041 31480563 PMC6770113

[37] Fekri Z Aghebati N Sadeghi T Farzadfard MT. The effects of abdominal “I LOV U” massage along with lifestyle training on constipation and distension in the elderly with stroke. Complement Ther Med. (2021) 57:102665. 10.1016/j.ctim.2021.102665 33465382

[38] Reis AMD Fruchtenicht AV Loss SH Moreira LF. Use of dietary fibers in enteral nutrition of critically ill patients: a systematic review. Rev Bras Ter Intensiva. (2018) 30:358–65. 10.5935/0103-507X.20180050 30328989 PMC6180475

[39] Reintam Blaser A Starkopf J Malbrain ML. Abdominal signs and symptoms in intensive care patients. Anaesthesiol Intensive Ther. (2015) 47:379–87. 10.5603/AIT.a2015.0022 25973664

[40] Stubley SJ Cayre OJ Murray BS Celigueta Torres I. Pectin-based microgels for rheological modification in the dilute to concentrated regimes. J Colloid Interface Sci. (2022) 628:684–695. 10.1016/j.jcis.2022.07.147 35944299

[41] Wen X Zhong R Dang G Xia B Wu W Tang S . Pectin supplementation ameliorates intestinal epithelial barrier function damage by modulating intestinal microbiota in lipopolysaccharide-challenged piglets. J Nutr Biochem. (2022) 109:109107. 10.1016/j.jnutbio.2022.109107 35863585

[42] Kopjar M Corkovic I Buljeta I Simunovic J Pichler A. Fortification of pectin/blackberry hydrogels with apple fibers: effect on phenolics, antioxidant activity and inhibition of alpha-glucosidase. Antioxidants. (2022) 11:1457. 10.3390/antiox11081459 35892661 PMC9332755

[43] Li Y Hou L Jiang E. Research progress on the application of semi-solidified enteral nutrition in critically ill patients. Nurs. Manage. China. (2023) 23:781–785.

[44] Patel JJ Rosenthal MD Heyland DK. Intermittent versus continuous feeding in critically ill adults. Curr Opin Clin Nutr Metab Care. (2018) 21:116–20. 10.1097/MCO.0000000000000447 29232262

[45] Reinhold S Yeginsoy D Hollinger A Todorov A Tintignac L Sinnreich M . Protein delivery in intermittent and continuous enteral nutrition with a protein-rich formula in critically ill patients-a protocol for the prospective randomized controlled proof-of-concept Protein Bolus Nutrition (Pro BoNo) study. Trials. (2020) 21:740. 10.1186/s13063-020-04635-1 32843075 PMC7449093

[46] Qu J Xu X Xu C Ding X Zhang K Hu L. The effect of intermittent versus continuous enteral feeding for critically ill patients: a meta-analysis of randomized controlled trials. Front Nutr. (2023) 10:1214774. 10.3389/fnut.2023.1214774 37671198 PMC10475573

[47] Huang T Liu Y Sun X. Comparison of two different enteral nutrition methods in critically ill patients. Chin Pharm. (2017) 29:4–6.

[48] Theodoridis X Chrysoula L Evripidou K Kalaitzopoulou I Chourdakis M. Continuous versus intermittent enteral feeding in critically ill children: a systematic review. Nutrients. (2023) 15:288. 10.3390/nu15020288 36678158 PMC9867148

[49] Heffernan AJ Talekar C Henain M Purcell L Palmer M White H. Comparison of continuous versus intermittent enteral feeding in critically ill patients: a systematic review and meta-analysis. Crit Care. (2022) 26:325. 10.1186/s13054-022-04140-8 36284334 PMC9594889

[50] Gonzalez JT Dirks ML Holwerda AM Kouw IWK van Loon LJC. Intermittent versus continuous enteral nutrition attenuates increases in insulin and leptin during short-term bed rest. Eur J Appl Physiol. (2020) 120:2083–94. 10.1007/s00421-020-04431-4 32651634 PMC7419443

[51] Li PF Wang YL Fang YL Nan L Zhou J Zhang D. Effect of early enteral nutrition on outcomes of trauma patients requiring intensive care. Chin J Traumatol. (2020) 23:163–7. 10.1016/j.cjtee.2020.04.006 32456954 PMC7296358

[52] Lu GY Xu H Li JH Chen JK Ning YG. Safety and outcome of early enteral nutrition in patients receiving extracorporeal membrane oxygenation. Clin Nutr. (2023) 42:1711–4. 10.1016/j.clnu.2023.07.021 37541102

[53] Haines KL Ohnuma T Grisel B Krishnamoorthy V Raghunathan K Sulo S . Early enteral nutrition is associated with improved outcomes in critically ill mechanically ventilated medical and surgical patients. Clin Nutr ESPEN. (2023) 57:311–7. 10.1016/j.clnesp.2023.07.001 37739674

